# Acute arterial occlusion of the lower limb as the main clinical manifestation in a patient with Covid-19 – Case Report

**DOI:** 10.1016/j.ijscr.2020.11.046

**Published:** 2020-11-11

**Authors:** Víctor de Oliveira Costa, Guilherme Bicalho Civinelli de Almeida, Eveline Montessi Nicolini, Guilherme de Abreu Rodrigues, Bruna Malaquias Arguelles da Costa, Guilherme Heluey Carvalho, Álvaro Luiz Segregio dos Reis, Davi Pinto Colen

**Affiliations:** a Faculdade de Ciências Médicas e da Saúde de Juiz de Fora and Universidade Federal de Juiz de Fora, Juiz de Fora, Minas Gerais, Brazil; b Vascular Surgery, Hospital Monte Sinai and Hospital Universitário da Universidade Federal de Juiz de Fora, Juiz de Fora, Minas Gerais, Brazil; c Thoracic Surgery, Hospital Monte Sinai, Juiz de fora, Minas Gerais, Brazil; d Faculdade de Ciências Médicas e da Saúde de Juiz de Fora, Juiz de Fora, Minas Gerais, Brazil; e Vascular Surgery, Hospital Monte Sinai and Santa Casa de Juiz de Fora, Juiz de Fora, Minas Gerais, Brazil; f Vascular Surgery, Hospital Monte Sinai and Unimed Juiz de Fora, Juiz de Fora, Minas Gerais, Brazil

**Keywords:** Thrombosis, Coronavirus infections, Ischemia, Case report

## Abstract

**Introdution:**

COVID-19 infection may predispose to venous and arterial thromboembolism due to excessive inflammation, hypoxia, immobilization and disseminated intravascular coagulation; however, there are few reports of lower limb ischemia as the main manifestation of the disease.

**Presentation of case:**

Male patient, 69 years old, asthmatic, ex-smoker and bearer of systemic arterial hypertension, has been admitted to the emergency department with sudden onset of pain in the right lower limb (RLL), associated with cyanosis and reduced temperature of the limb. He has been tested for COVID-19 in the OR with positive result for IGG and IGM. Computed tomography angiography (AngioCT) was performed, showing signs of arterial embolization to both limbs, right internal iliac artery, and superior mesenteric artery. Faced with the threat of limb loss and the absence of signs and symptoms of visceral ischemia, the patient underwent full anticoagulation and RLL thromboembolectomy and tricompartmental fasciotomy. He was discharged after 7 days of hospitalization and demonstrated no other signs and symptoms of COVID-19, following outpatient follow-up.

**Discussion:**

COVID-19 is associated with high risk of thrombotic complications being related to the clinical severity of the patient, with few studies that show symptoms of sudden pain in the lower limb without other complaints.

**Conclusion:**

Individuals infected with COVID-19 are at risk for arterial thromboembolic events, and knowledge of such cases is essential in order to create specific protocols for prophylaxis of thrombotic events in these patients, in addition to increasing the suspicion of infection in individuals with acute arterial occlusion, mostly during pandemic times.

## Highlights

• Thrombotic complications have been increasingly observed in patients with COVID-19.
• Confirming IGG and IGM + by immunochromatographic method.
• AngioCT showing signs of arterial embolization to both limbs.
• The patient underwent in the right lower limb thromboembolectomy.
• Tricompartmental fasciotomy was performed to prevent compartment syndrome.

## 1 Introduction

Coagulopathies and thrombotic complications have been increasingly observed in patients with severe disease [[1], [2], [3], [4]]. Several necropsy studies have revealed platelet-fibrin buffers in pulmonary arterioles. It has been suggested that the thrombotic diathesis associated with COVID-19 reflects an endotheliopathy induced by a viral infection of endothelial cells. These cells prominently express the plasma membrane protein ACE2 to which the spike protein of the SARS-CoV-2 virions binds, enabling their endosomal incorporation into the cells. The thrombotic complications of COVID-19 infection would be readily explained if the virus had infected endothelial cells and induced luminal expression of tissue factor (TF), which could then interact with circulating coagulation factor VII to trigger a proteolytic cascade culminating in generation of thrombin and fibrin (extrinsic coagulation). TF expression is negligible in healthy non-inflamed endothelial cells, but it can be increased in transcription level by various pro-inflammatory stimuli that activate the NF-kappaB transcription factor [5].

In view of the relation between thromboembolic events and patients infected with COVID-19, the need of protocols for the prevention of thrombotic events in these patients is evident, as well as the suspicion of COVID-19 infection in patients who present the thrombotic or thromboembolic event as the first symptom. The report has been arranged in line with SCARE guidelines [6].

## 2 Presentation of case

**Fig. 1:**
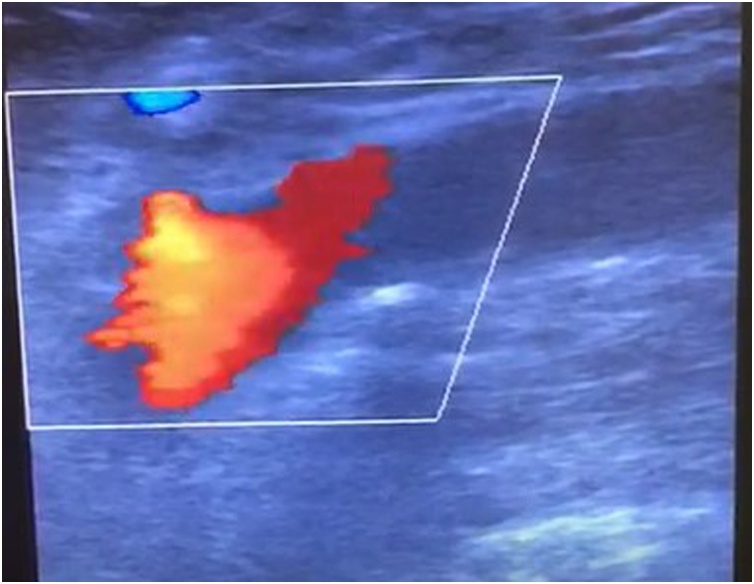
Doppler showing common femoral occlusion.

A 69-year-old male patient brought by the Emergency Medical Service during the COVID-19 epidemic, asthmatic onset at the age of 20, former smoker for 20 years, frequently using Salbutamol, with systemic arterial hypertension (SAH) taking losartan and atenolol. He stated that 6 h before hospital admission he started to feel a sudden pain in the RLL, associated with cyanosis of the limb and decreased temperature. On physical examination, he presented regular heart rhythm and absence of pulses in the RLL, with painful and limited movement, cold limb from the middle third of the leg to the toes, and no swelling, with the left lower limb having palpable and wide pulses with no evidence of previous peripheral artery disease. The patient reported having had pneumonia-like symptoms in the previous week with partial improvement after antibiotic therapy with Levofloxacin, in addition to the concomitant use of oseltamivir and edoxaban. The hypothesis of COVID-19 was raised and a test performed in the OR, confirming IGG and IGM + by immunochromatographic method. Still in the emergency, arterial Doppler echocardiography was performed, showing a thrombus in the right common femoral artery (Fig. 1).

**Fig. 2:**
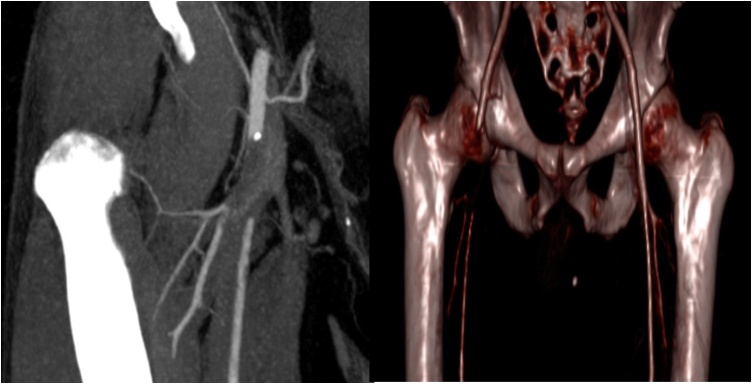
Image showing occlusion of the common femoral artery and origin of the superficial femoral artery and deep superficial artery.

Thus, angioCT of the abdominal aorta, iliac arteries and lower limb arteries was requested, with occlusion of the common femoral artery and small proximal portions of the right superficial and deep femoral arteries, occlusion of the proximal portion of the right anterior tibial artery, occlusion of the tibio-fibular trunk and proximal portions of the left posterior tibial and fibular arteries, subocclusion of the right tibial-fibular trunk, occlusion of the proximal portion of the superior mesenteric artery (SMA) and occlusion of the right internal iliac artery (Fig. 2).

**Fig. 3:**
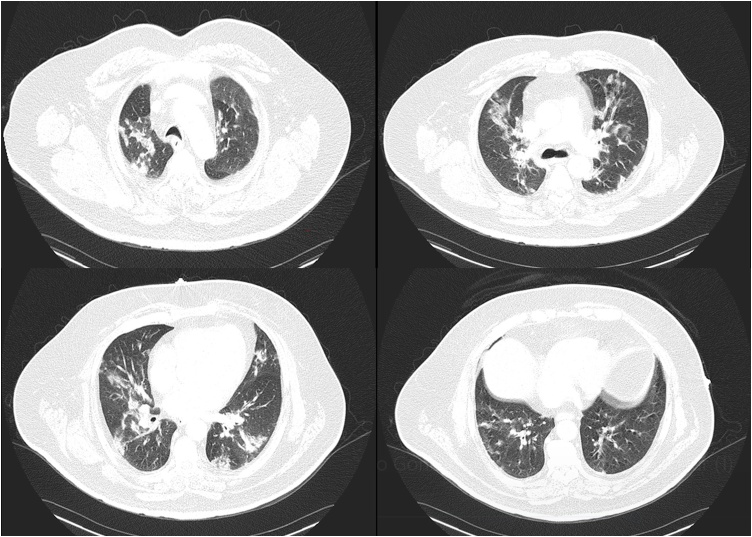
Typical tomographic findings for viral pneumonia, with involvement in 25% and 50% of the lung parenchyma. Small calcified plaque in the aortic arch and more exuberant fibrous atheromatous plaque in the descending thoracic aorta.

In addition, a chest CT was taken at the same time as the angioCT, which evidenced findings that would suggest COVID-19 with multiple focal ground-glass opacities, creating consolidations, parse in both lungs, some with linear / reticular opacities, affecting between 25% and 50% of the lung parenchyma. The patient underwent emergency surgery for limb salvage, as he demonstrated no abdominal symptoms despite the SMA occlusion. The patient underwent RLL thromboembolectomy, with a large quantity of fresh thrombi coming out of the common, superficial, and deep femoral arteries, presenting a pedal pulse at the end of the surgery. Tricompartmental fasciotomy was performed to prevent reperfusion compartment syndrome. The procedure was performed by Doctor Guilherme Bicalho Civinelli de Almeida and by Doctor Guilherme Heluey Carvalho, both specialized in Vascular Surgery, Angioradiology and Endovascular Surgery (Fig. 3).

After the procedure, the patient was admitted to the COVID-19 ICU, due to pneumonia-like symptoms on the previous week and a positive covid-19 test. During hospitalization, he developed renal dysfunction, being treated with conservative measures. He presented progressive improvement of renal function, without complications in the lower limb, being transferred to the hospitalization unit for observation, after 5 days in the ICU, since he did not present respiratory symptoms and peripheral oxygen saturation above 94%. The patient evolved without additional complications, being submitted to fasciotomy synthesis on the seventh postoperative day, and hospital discharge on the eighth postoperative day.

**Fig. 4:**
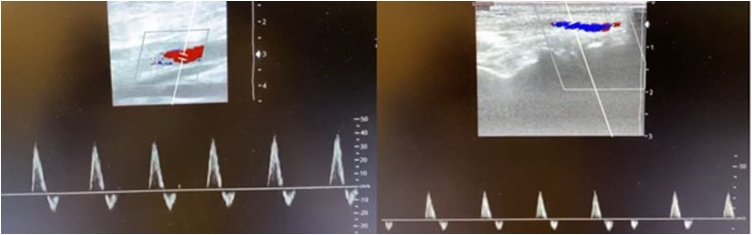
Image showing biphasic flow echo-Doppler in right lower limb arteries.

The patient was discharged with the anticoagulant Edoxaban and medical staff opted for prolonged anticoagulation for at least one year. After 3 months of follow-up there were no other complications and a biphasic flow was present in the arteries of the RLL, as evidenced by a control duplex scan (Fig. 4).

## 3 Discussion

Cui S et al. reported frequent venous thrombotic episodes in severe cases of COVID-19, among which survival was longer with heparin thromboprophylaxis [7]. In addition, another study observed frequent venous thromboembolic complications in COVID-19, with a risk that appears particularly high in patients requiring admission to the ICU and/or with obesity, with complications due to thrombosis such as frequent clotting of catheters, dialysis filters and ECMO oxygenators and arterial thrombotic events, including acute ischemia or stroke [8]. Besides, pulmonary embolism has recently been identified as the most common thrombotic event in these patients, which occurs despite thromboprophylaxis [9]. However, no study has formally documented increased risk of thrombosis in COVID-19 compared to other serious infections, nor demonstrated that this risk was associated with a poor prognosis [10].

A review found that the infection by COVID-19 is associated with a high risk of thrombotic complications, with the occurrence of venous thromboembolism and stroke in approximately 20% and 3% of patients, respectively. In addition, a higher frequency of these events was found in critically ill patients, especially those admitted to the ICU, despite the use of anticoagulant prophylaxis [11].

Klok et al. described 184 patients admitted with symptoms of COVID-19 to the intensive care unit. The cumulative incidence of thrombotic disorders was 31%. AngioCT or duplex ultrasound was used to diagnose deep vein thrombosis (DVT), which was detected in 27% and arterial thrombotic events in 3.7%. Pulmonary embolism was the most common thrombotic complication (81%) [12]. In another study by Zhou et al, a patient with COVID-19 showed symptoms of acute cerebral infarction, with this and DVT being confirmed in both lower limbs after angioCT [13]. There is a report of a COVID-19 patient having complications with venous thrombosis of both lower limbs, occlusion of the anterior tibial artery and occlusion of the dorsalis pedis artery of both lower limbs, resulting in death subsequently due to acute respiratory failure [14].

In another case report, a 57-year-old patient who presented edema, pain, heat and redness in the left lower limb (LLL) was described, having been treated with heparin. The LLL venous doppler revealed dilation and thrombosis in the external iliac vein up to the bifurcation level of the common iliac veins, in addition to thrombosis of the great and small saphenous vein, implying a DVT predisposition resulting from COVID-19 [15]. In another case, a nonsmoker 48-year-old male patient, with a history of coronary artery disease, was hospitalized with COVID-19, having received prophylaxis for thrombosis with heparin, returned to the hospital two weeks later with pain in his right lower limb, being diagnosed with DVT in femoral, popliteal and gastrocnemic veins, which suggested the prothrombotic condition present in COVID-19 infections [16].

In a report similar to our case, a 68-year-old patient with a history of smoking, SAH, coronary artery disease and deep vein thrombosis was admitted for acute bilateral ischemia of the lower limbs, in which obstructive thrombosis of the abdominal aorta and bilateral thrombosis of the common iliac arteries. Axillobifemoral bypass was performed, followed by therapeutic anticoagulation with good initial results, but the patient died after seven days due to severe bleeding [17]. In another case report, a 60-year-old patient went to the hospital with sudden numbness and loss of strength in both legs. Before that, he reported a dry cough, fever and general malaise for 2 weeks before the hospital presentation, but no pre-existing symptoms of peripheral vascular disease. AngioCT revealed an acute thrombotic occlusion of the infrarenal aorta that extended to the common iliac arteries. The patient underwent thromboembolectomy with retrieval of a large burden of acute thrombus. He had a good outcome after surgery and was discharged [18]. In recent evidence, the involvement of arterial beds is usually associated with hospitalization for longer periods or patients in the ICU, being related to the clinical severity of the patient, with few studies that show symptoms of sudden pain in the lower limb without other complaints, as seen in our report [19,20].

## 4 Conclusion

This case aims to raise the awareness of the medical community regarding thrombotic events as a clinical symptom of COVID-19, even if the patient does not have typical symptoms of COVID-19, demanding immediate treatment in order to reduce complications resulting from these events. In addition, it is notable that even patients receiving prophylaxis for thrombosis can develop this condition, which demonstrates the need for specific protocols for venous and arterial prophylaxis in patients with COVID-19.

## Declaration of Competing Interest

No conflict of interest.

## Funding

No fund to my research to be disclosed.

## Ethical Approval

This is case report study and ethical approval not required.

## Consent

Written informed consent was obtained from the patient for publication of this case report and accompanying images. A copy of the written consent is available for review by the Editor-in-Chief of this journal on request.

## Author contribution

Dr. Víctor de Oliveira Costa (literature review, case description, discussion and conclusion).

Dr. Guilherme Bicalho Civinelli de Almeida (case description, discussion, conclusion and Surgical procedure).

Dr. Eveline Montessi Nicolini (collected the images from the patient file and collected the patient history).

Dr. Guilherme de Abreu Rodrigues (collected the patient history and examination from the file and he wrote the references).

Bruna Malaquias Arguelles da Costa (literature review, collected the patient history and examination from the file and she wrote the references).

Dr. Guilherme Heluey Carvalho (Case description, Surgical procedure).

Dr. Álvaro Luiz Segregio dos Reis (Case description).

Dr.Davi Pinto Colen (Case description).

## Registration of Research Studies

1. Name of the registry: Not applicable.
2. Unique identifying number or registration ID: Not applicable.
3. Hyperlink to your specific registration (must be publicly accessible and will be checked): Not applicable.

## Guarantor

Dra. Eveline Montessi Nicolini.

## Provenance and peer review

Not commissioned, externally peer-reviewed.
